# Sex-dependent associations between *MAP3K1* gene polymorphisms and soy products with the gastric cancer risk in Korea: a case-control study

**DOI:** 10.1186/s12876-022-02569-3

**Published:** 2022-12-12

**Authors:** Jung Hyun Kwak, Chang Soo Eun, Dong Soo Han, Yong Sung Kim, Kyu Sang Song, Bo Youl Choi, Hyun Ja Kim

**Affiliations:** 1 Department of Food and Nutrition, Gangneung-Wonju National University, 7 Jukheon-gil, Gangneung-si, Gangwon-do 25457 Korea; 2 Division of Gastroenterology, Department of Internal Medicine, Hanyang University Guri Hospital, Guri-si, Korea; 3 Functional Genomics Institute, PDXen Biosystems Co., ETRI Convergence Commercialization Center, Daejeon, Korea; 4 Department of Pathology, Chungnam National University College of Medicine, Daejeon, Korea; 5 Department of Preventive Medicine, Hanyang University College of Medicine, 222 Wangsimni-ro, Seongdong-gu, Seoul, 04763 Korea

**Keywords:** Case-control studies, Stomach Neoplasms, Soy products, *MAP3K1*, Polymorphism, Single Nucleotide, Diet

## Abstract

**Background/Objectives:**

The hormone-dependent effect of *MAP3K1* gene polymorphisms may explain sex-specific differences in gastric cancer (GC) risk. Phytoestrogens have been shown to interact with this genetic factor. Here, we investigated the association between *MAP3K1* gene polymorphisms and GC risk by sex and whether these associations differ depending on soy products intake.

**Methods:**

Participants aged 20–79 years were recruited from two hospitals between December 2002 and September 2006. In all, 440 cases and 485 controls were recruited, among, 246 pairs of cases and controls, matched by sex, age (± 5 years), study admission period (± 1 years), and hospital, were included for the analysis.

**Results:**

In dominant model, men with the A allele of rs252902 showed significantly increased GC risk (odd ratio; OR=2.19, 95% confidence interval; CI=1.31–3.64) compared to GG homozygotes. When stratified by intake of soy products, men with the A allele of rs252902 and low intake of soy products showed significantly higher GC risk (OR=3.29, 95% CI=1.55–6.78) than that in GG homozygotes.

**Conclusions:**

Men with the risk allele of *MAP3K1* had a significantly increased GC risk compared to GG homozygotes; this trend was more pronounced in those with low intake of soy products.

**Supplementary Information:**

The online version contains supplementary material available at 10.1186/s12876-022-02569-3.

## Introduction

According to GLOBOCAN 2020, gastric cancer (GC) is the fifth most common cancer worldwide, with the highest incidence rate in Eastern Asia [1]. In Korea, GC is the most prevalent cancer in men aged 35–64 years old [2], and the incidence rate is approximately two-fold higher in men than in women [3]. The difference in GC incidence may be due to differences in behavioral factors, such as alcohol consumption, smoking, and poor eating habits (e.g., high salt intake) [3], as well as differences in biological factors, such as sex hormones [4] and genetic predisposition [5]. Indeed, several studies have reported that the female hormone estrogen has a protective effect against GC risk [4, 6]. Genetic predisposition is estimated to affect the GC risk by 3–28% [7, 8], and the distribution of specific gene mutations (e.g., *Germline E-cadherin*) differs according to sex [9]. The mitogen-activated protein kinase kinase 1 (*MAP3K1*) gene encodes a serine/threonine kinase that acts on the nuclear factor kappa B (NF-κB) pathway [10]. NF-κB acts as a regulator of the immune system and inflammatory response by upregulating cytokines/chemokines [11]. Elevated NF-κB activity and increased levels of pro-inflammatory cytokines have been reported in tumorous tissues [12]. Indeed, Yang et al. (2012) reported that participants with a risk allele in the *MAP3K1* gene (rs16886448 and rs252902) showed significantly higher GC risk [5]. Fortunately, several dietary factors (i.e., an antioxidant based diet or soy products including isoflavones) have been reported to interact with genetic factors that affect GC risk [13, 14]. In particular, soy products are abundant in phytoestrogens such as genistein, daidzein, and glycitein [15], which can mimic the function of estrogen. Siriviriyakul et al. (2020) reported that genistein has gastroprotective effects through the reduction of pro-inflammatory mediators and nuclear receptor NF-κB expression in an animal model [16]. Additionally, Yang et al. (2012) [5] demonstrated that the gene polymorphism of *MAP3K1* also interacts with enterolactone, a phytoestrogen [5]. They suggested that the hormone-dependent effect of the *MAP3K1* gene polymorphism may explain the sex-specific differences in GC risk. However, no study has investigated the association between *MAP3K1* gene polymorphisms and GC risk according to sex. Previous studies have focused on the association between *MAP3K1* and hormone-related cancers such as breast cancer [17, 18].

Therefore, the primary goal of the present study was to determine the association between *MAP3K1* gene polymorphisms and GC risk by sex. The secondary goal of this study was to evaluate whether the combined association between soy products intake and *MAP3K1* gene polymorphisms alters the GC risk through a case-control study in Korea.

## Materials and Methods

### Study population

Participants aged 20–79 years were recruited from two hospitals (Chungnam University Hospital and Hanyang University Guri Hospital) between December 2002 and September 2006. GC cases were histologically diagnosed based on the World Health Organization classification of tumors of the digestive system as follows [19]: gastroscopy was performed by a gastroenterologist, and the final diagnosis of GC was confirmed by a pathologist through a biopsy. Controls were participants without any gastric problems who were recruited from the same hospitals during the same period. A total of 440 cases and 485 controls were included in this study. Among them, the results showed that 324 cases and 276 controls had SNP (rs252902) information. However, participants who were not analyzed owing to low DNA concentrations (cases=5, controls=6) and participants with abnormal energy intake (<500 kcal; case=1, controls=2 or >5000 kcal; case=1, controls=7) were excluded. Age within five years, sex, registered hospitals, and study admission period within one year were matched in a 1:1 ratio in 317 cases and 261 controls. Ultimately, 246 pairs were included in the analysis. All participants provided written informed consent, and the study protocol was approved by the Institutional Review Board of Hanyang University Medical Center (IRB no. 2003-4). Our research was performed in accordance with relevant guidelines/regulations, and performed in accordance with the Declaration of Helsinki. Informed consent was obtained from all participants.

### Data collection

A questionnaire-based survey was conducted by trained interviewers to collect information on sociodemographic characteristics, behavioral factors, disease status, family history, and dietary habits. Data on dietary factors were collected by using a quantitative food frequency questionnaire (FFQ). We used a slightly modified version of the validated FFQ used in our previous study [20]. A detailed description of the dietary data collection in this study has been previously published [21]. Total energy intake was calculated using the Korean Foods and Nutrients Database [22]. The items related to the intake of noodles and dumplings was composed of eight dishes which were: 1. Noodles, banquet noodles (janchi-guksu), cold noodles (naengmyeon), and noodles in a cold soybean soup (kong-guksu); 2. Spicy mixed noodles, spicy noodles (bibim-guksu), spicy cold chewy noodles (jjolmyeon), and cold noodles with spicy dressing (bibim-naengmyeon); 3. Instant noodles (ramen); 4. Noodles with black bean sauce (Jajangmyeon); 5. Chinese-style noodles with vegetables and seafood (jjamppong); 6. hand-pulled dough soup (Sujebi), chopped noodles (Kal-guksu); 7. Dumplings and dumpling soups; 8. Sliced rice cake soup (teok-guk). The items related to the intake of soy products was divided into three foods: 1. Soybeans that were boiled in soy sauce (kongjaban); 2. Tofu; 3. Soymilk. The daily intake of soy products, noodles and dumpling was calculated by multiplying the frequency per day and the intake amount, and then adding them to estimate the daily intake.

### Genotyping and SNP selection

In our study, we screened candidate genes associated with GC and selected two 2 SNPs (rs16886448 and rs252902) related to *MAP3K1*. Among them, the tag SNP rs252902, representing the *MAP3K1* gene, was finally selected and analyzed in our study. Before genotyping, genomic DNA was extracted by isolating peripheral blood leukocytes from the participants’ whole blood. The DNA concentration was determined using a NanoDrop ND-1000 spectrophotometer for primary quality control (Thermo Fisher Scientific Inc., Wilmington, U.S.). SNP genotyping was performed using the KASP Assay [23] with a QuantStudio 5 Real-Time PCR Instrument, according to the manufacturer’s protocol (Thermo Fisher Scientific Inc., Wilmington, U.S.). The genotyping results confirmed quality control through minor allele frequency (MAF) and Hardy–Weinberg equilibrium (HWE). There was no genotyping error when the MAF was more than 1%, the p-value of HWE was > 0.05, and the SNP call rate > 95%.

### Statistical analysis

All statistical analyses were conducted using SAS version 9.4 (SAS Institute Inc., Cary, NC, USA). The general characteristics of the participants were compared between the cases and controls by sex using Student’s *t*-test for continuous variables (mean ± SD) and chi-squared test for categorical variables (n, %). The odds ratio (OR) and 95% confidence interval (CI) for GC risk were calculated using logistic regression after controlling for known risk factors. The genetic polymorphism of GC was assessed under the dominant and recessive model. The interactions between the SNP and sex were analyzed by a likelihood ratio test, where the multiplicative interaction term of the SNP and sex was added to the logistic regression model. To analyze the combined associations of soy products with genetic polymorphisms, low or high intake of soy products was categorized according to the median intake of the controls. We adjusted for the following covariates in the model: age (as continuous), BMI (≤22.99, ≥ 23.0, or missing), education level (≤ middle school, ≥ high school, or missing), family history (first-degree relatives) of GC (no or yes), smoking status (never, past, or current smoker), alcohol consumption (never, past, or current drinker), hospitals (Chungnam University Hospital or Hanyang University Guri Hospital), *H. pylori* infection (negative, positive, or undetermined), daily intake of noodles and dumplings (as continuous), and total energy intake (as continuous).

## Results

### General characteristics of the GC cases and controls according to sex

Table 1 shows the general characteristics of the GC cases and controls according to sex. In men, the mean ages of the cases and controls were 57.8 (±10.8) years and 56.7 (±11.0) years, respectively. The cases had more former and current smokers (90.2%) than the controls (81.7%) (p = 0.026). The cases had a higher proportion of underweight and normal weight individuals (50.0%) than the controls (34.2%) (p = 0.014). The controls had a higher proportion of participants with *H. pylori* infection (48.2%) than the cases (31.7%) (p = 0.009). The cases consumed significantly more total energy, and daily intake of noodles and dumplings than the controls. However, no difference was observed between cases and controls based on education level, alcohol consumption, registered hospitals, family history of GC, and daily intake of soy products.

**Table 1:** Participant characteristics of GC cases and controls according to sex

Characteristics	Men (*n*=328)					Women (*n*=164)				
	Cases (*n*=164)		Controls (*n*=164)		*p*-values	Cases (*n*=82)		Controls (*n*=82)		*p*-values
Age (y, mean ± SD)	57.8	± 10.8	56.7	±11.0	0.353	55.9	±13.7	56.1	±12.9	0.916
Education level (*n*, %)										
≤ Middle school	66	(40.2)	61	(37.2)	0.802	45	(54.9)	45	(54.9)	0.959
≥ High school	82	(50.0)	88	(53.7)		29	(35.4)	30	(36.6)	
Missing	16	(9.8)	15	(9.1)		8	(9.7)	7	(8.5)	
Smoking status (*n*, %)										
Never	16	(9.8)	30	(18.3)	0.026	70	(85.4)	76	(92.7)	0.134
Former & Current	148	(90.2)	134	(81.7)		12	(14.6)	6	(7.3)	
Alcohol consumption (*n*, %)										
Never	28	(17.1)	27	(16.5)	0.883	56	(68.3)	56	(68.3)	1.000
Former & Current	136	(82.9)	137	(83.5)		26	(31.7)	26	(31.7)	
Body mass index (kg/m^2^) (*n*, %)										
≤ 22.99	82	(50.0)	56	(34.2)	0.014	45	(54.8)	32	(39.0)	0.122
≥ 23.0	74	(45.1)	99	(60.4)		29	(35.4)	38	(46.4)	
Missing	8	(4.9)	9	(5.5)		8	(9.8)	12	(14.6)	
Hospital (*n*, %)										
Chungnam university	58	(35.4)	58	(35.4)	1.000	28	(34.2)	28	(34.2)	1.000
Hanyang university Guri	106	(64.6)	106	(64.6)		54	(65.8)	54	(65.8)	
Family history of gastric cancer (*n*, %)^a^										
No	138	(84.1)	148	(90.2)	0.098	72	(87.8)	74	(90.2)	0.617
Yes	26	(15.9)	16	(9.8)		10	(12.2)	8	(9.8)	
*H. pylori* infection (*n*, %)										
No	58	(35.4)	43	(26.2)	0.009	29	(35.4)	13	(15.9)	0.017
Yes	52	(31.7)	79	(48.2)		24	(29.2)	31	(37.8)	
Undetermined	54	(32.9)	42	(25.6)		29	(35.4)	38	(46.3)	
*MAP3K1 rs252902* polymorphisms (*n*, %)										
GG	45	(27.4)	73	(44.5)	0.004	33	(40.3)	28	(34.2)	0.529
GA	81	(49.4)	67	(40.9)		37	(45.1)	37	(45.1)	
AA	38	(23.2)	24	(14.6)		12	(14.6)	17	(20.7)	
Daily intake of dietary factor (mean ± SD)										
Total energy intake (kcal/d)	2011	±760	1770	±644	0.002	1509	±516	1593	±485	0.285
Noodle and dumpling (g/day)	92.2	±137.8	60.1	±64.5	0.008	44.6	±43.4	43.2	±47.9	0.841
Soy products (g/day)	19.3	±35.3	16.6	±30.6	0.464	22.1	±72.4	16.3	±33.8	0.508

^a^first-degree relatives. Based on Student’s t-test for continuous variables, or chi-squared test for categorical variables

In women, the mean ages of the cases and controls were 55.9 (±13.7) years and 56.1 (±12.9) years, respectively. The controls had a higher proportion of participants with *H. pylori* infection (37.8%) than the cases (29.2%) (p = 0.017). There were no significant differences in the other covariates (education level, smoking, alcohol consumption, and daily intake of soy products, etc.).

### Associations between the general characteristics and GC risk by sex

Table 2 shows the association between the general characteristics and GC risk according to sex. In men, there was a significantly higher GC risk (OR = 2.40, 95% CI = 1.13–5.09) in those who were former and current smokers, and significantly lower GC risk (OR = 0.45, 95% CI = 0.27–0.75) in those who were overweight and obese. However, there were no significant differences between the general characteristics and GC risk in women.

**Table 2:** Associations of general characteristics with GC risk according to sex

Variables	Men		Women	
	OR	(95 % CI)^a^	OR	(95 % CI)^a^
Education level				
≤ Middle school	1.00	Ref.	1.00	Ref.
≥ High school	0.82	(0.48-1.42)	0.93	(0.36-2.40)
Smoking status				
Never	1.00	Ref.	1.00	Ref.
Former & current	2.40	(1.13-5.09)	2.60	(0.85-7.92)
Alcohol consumption				
Never	1.00	Ref.	1.00	Ref.
Former & current	1.05	(0.56-1.97)	0.86	(0.40-1.85)
Body mass index (kg/m^2^)				
Underweight & Normal weight (≤ 22.99)	1.00	Ref.	1.00	Ref.
Overweight & Obesity (≥ 23.0)	0.45	(0.27-0.75)	0.52	(0.26-1.07)
Family history of gastric cancer				
No	1.00	Ref.	1.00	Ref.
Yes	1.57	(0.77-3.22)	1.28	(0.44-3.72)

OR, odds ratios; CI, confidence interval.

^a^Adjusted for age, sex, body mass index (≤ 22.99, or ≥ 23.0), education level (≤ middle school, ≥ high school, or missing), family history of gastric cancer (no or yes), smoking status (never, past and current smokers), alcohol consumption (never, past and current), hospital (Chungnam university hospital or Hanyang university Guri hospital), *H. pylori* infection (negative, positive, or undetermined), daily intakes of noodles and dumpling, and total energy intake (continuous).

### Associations between *MAP3K1* gene polymorphisms with GC risk by sex

Table 3 shows the association between the *MAP3K1* gene polymorphism and GC risk. In men, those with the *MAP3K1* rs252902 polymorphism showed a significant increase in the GC risk in the GA type (OR = 2.02, 95% CI = 1.17–3.47) or AA type (OR = 2.67, 95% CI = 1.33–5.35) when compared to that seen in the GG homozygotes. In the dominant model, men with the A allele of *MAP3K1* rs252902 showed a significant increase in the GC risk (OR = 2.19, 95% CI = 1.31–3.64) when compared to that seen in GG homozygotes. In women, there was no statistically significant association between the *MAP3K1* rs252902 polymorphism and GC risk. The interaction between *MAP3K1* rs252902 and sex was significant (p < 0.05).

**Table 3:** Associations of *MAP3K1* rs252902 polymorphism with the risk of GC risk

	Men			Women		
Variables	No. of cases/controls	OR	(95 % CI)^a^	No. of cases/controls	OR	(95 % CI)^a^
*MAP3K1 rs252902*^b^						
GG	45/73	1.00	Ref.	33/28	1.00	Ref.
GA	81/67	2.02	(1.17-3.47)	37/37	0.89	(0.41-1.90)
AA	38/24	2.67	(1.33-5.35)	12/17	0.52	(0.20-1.37)
Dominant model						
GG	45/73	1.00	Ref.	33/28	1.00	Ref.
GA+AA	119/91	2.19	(1.31-3.64)	49/54	0.75	(0.37-1.52)
Recessive model						
GG+GA	126/140	1.00	Ref.	70/65	1.00	Ref.
AA	38/24	1.78	(0.96-3.29)	12/17	0.55	(0.23-1.38)

OR, odds ratios; CI, confidence interval.

^a^Adjusted for age, sex, body mass index (≤ 22.99, or ≥ 23.0), education level (≤ middle school, ≥ high school, or missing), family history of gastric cancer (no or yes), smoking status (never, past and current smokers), alcohol consumption (never, past and current), hospital (Chungnam university hospital or Hanyang university Guri hospital), *H. pylori* infection (negative, positive, or undetermined), daily intakes of noodles and dumpling, and total energy intake (continuous).

^b^The *P*-value for interaction was significant (*P* <0.05)

### Association between *MAP3K1* gene polymorphisms and GC risk stratified by intake of soy products

Table 4 shows the associations between *MAP3K1* gene polymorphisms and GC risk stratified by soy products intake. For total intake of soy products, women with high intake of soy products tended to exhibit lower GC risk (OR = 0.49, 95% CI = 0.24–1.01) when compared to that in women with low intake of soy products. When we stratified by intake of soy products and the *MAP3K1* gene polymorphism, men with the A allele of *MAP3K1* rs252902 and low intake of soy products showed significantly higher GC risk (OR = 3.29, 95% CI = 1.55–6.78) than that seen in GG homozygotes. This association was not significant among men with the A allele of *MAP3K1* rs252902 with high intake of soy products. In women, there was no statistically significant association between the *MAP3K1* rs252902 polymorphism and GC risk by soy product intake.

**Table 4:** Associations of *MAP3K1* rs252902 polymorphism with the GC risk according to intakes of soy products

	Men			Women		
Variables	No. of cases/controls	OR	(95 % CI)^a^	No. of cases/controls	OR	(95 % CI)^a^
Total intakes of soy products (g/day)						
Low	77/80	1.00	Ref.	55/39	1.00	Ref.
High	87/84	0.98	(0.59-1.63)	27/43	0.49	(0.24-1.01)
Low intakes of soy products						
GG	17/39	1.00	Ref.	23/17	1.00	Ref.
GA+AA	60/41	3.29	(1.55-6.78)	32/22	1.17	(0.42-3.27)
High intakes of soy products						
GG	28/34	1.00	Ref.	10/11	1.00	Ref.
GA+AA	59/50	1.50	(0.68-3.31)	17/32	0.38	(0.11-1.36)

OR, odds ratios; CI, confidence interval.

^a^Adjusted for age, sex, body mass index (≤ 22.99, or ≥ 23.0), education level (≤ middle school, ≥ high school, or missing), family history of gastric cancer (no or yes), smoking status (never, past and current smokers), alcohol consumption (never, past and current), hospital (Chungnam university hospital or Hanyang university Guri hospital), *H. pylori* infection (negative, positive, or undetermined), daily intakes of noodles and dumpling, and total energy intake (continuous)

## Discussion

Our findings have shown that men with the A allele of *MAP3K1* rs252902 had a significantly increased GC risk compared to that seen in GG homozygotes, and this association was more pronounced in those with a low intake of soy products.

In Korea, the incidence of GC is approximately two times higher in men than in women [3]. The difference in the GC incidence is likely attributable to differences in exposure to GC risk factors, such as alcohol consumption, smoking, and poor eating habits (e.g., high salt intake) [3]. In addition, biological differences in sex hormones are thought to affect GC incidence [4], as well as various diseases [24–26].

In a previous study, Yang et al. (2012) suggested that the hormone-dependent effect of *MAP3K1* gene polymorphisms (rs16886448 and rs252902) may explain sex-specific differences in the GC risk [5]. However, although several studies have focused on the association between *MAP3K1* polymorphisms and hormone-related cancers such as breast cancer [17, 18], no studies have confirmed the association between GC risk and this gene polymorphism by sex. *MAP3K1* is a serine-threonine kinase that acts in the mitogen-activated protein kinase (*MAPK*) pathway, which is a key mediator of cell physiology [10]. In addition, *MAP3K1* is thought to be involved in cell survival, apoptosis, and migration in normal and tumor cells. It is thought that *MAP3K1* affects GC because it acts on the NF- κB pathway [10]. NF- κB acts as a regulator of the immune system and inflammatory response by upregulating cytokines/chemokines [11]. Elevated NF-κB activity and increased expression of pro-inflammatory cytokines have been reported in tumor tissues [12]. Chronic inflammation increases cellular oxidative stress and DNA damage, which may lead to tissue damage and the initiation of cancer.

Several dietary factors (i.e., an antioxidant based diet or soy products including isoflavones) have been reported to interact with genetic factors affecting GC risk [13, 14, 27]. Kim et al. (2020) reported that the polymorphism of glutathione S-transferase Pi (*GSTP1*) rs1871042 increases the risk of GC (OR= 1.55, 95% CI = 1.10–2.16) [13]. However, they found that an antioxidant-rich diet might reduce the risk of GC according to the rs1871042 polymorphism. In addition, Yang et al. (2017) demonstrated that participants with a minor allele of *IL2* rs2069762 and a higher intake of non-fermented soy food had lower GC risk when compared to participants with different genetic characteristics [14]. Cho et al. (2015) reported that *NQO1* gene polymorphism in the ornithine decarboxylase-polyamine pathway is a genetic factor affecting GC, and an interaction with phytoestrogens such as genistein and daidzein could modify GC risk [27]. Yang et al. (2012) reported that the gene polymorphism of *MAP3K1* also interacts with enterolactone, a phytoestrogen [5]. They found that the C allele of *MAP3K1* rs16886448 was associated with an increased risk of GC at low enterolactone levels. In contrast, the A allele of *MAP3K1* rs252902 showed a significantly decreased risk of GC at high enterolactone levels. Similar to this previous study, we have found that men with the A allele of rs252902 had a significantly increased GC incidence when compared to GG homozygotes, and this association was more pronounced in those with a low intake of soy products. However, in contrast to previous studies, our study showed that this association was only observed in men, and we investigated whether the association differed according to the daily intake of soy products using the FFQ.

Soy products contain abundant cancer-preventive phytoestrogens such as genistein, daidzein, and glycitein [15]. Several studies have reported that the female hormone estrogen has a protective effect against GC risk [4, 6]. In an animal model, Siriviriyakul et al. (2020) reported that genistein had gastroprotective effects through the reduction of pro-inflammatory mediators and nuclear receptor NF-κB expression [16]. Therefore, it is thought that a high intake of soy products may help suppress GC incidence caused by NF-κB.

In our study, the association between *MAP3K1* and GC risk was not confirmed in women. We thought that this result might be due to the effects of female hormones. Thus, we additionally investigated the association between *MAP3K1* gene polymorphism and GC risk according to soy product intake in women aged 50 years and above, when the body estrogen concentration decreased. Although there was no statistical significance, the OR value of GC risk (OR = 1.61, 95% CI = 0.38–6.83) increased in women with the A allele of *MAP3K1* rs252902 and a low intake of soy products, whereas the OR value of GC risk (OR = 0.37, 95% CI = 0.05–2.60) decreased in women with the A allele of *MAP3K1* rs252902 and a high intake of soy products (Supplemental Table 1). However, since the sample size of the women was smaller than that of men, future studies with a larger sample size are needed to confirm the association between *MAP3K1* gene polymorphism and GC. Further experimental studies are needed to identify the unclear mechanisms for dietary factors that interact with these associations.

This study has several strengths. We have confirmed the association between *MAP3K1* gene polymorphism and GC by sex, and these associations may differ with the intake of soy products, which has not been reported in other studies before. To minimize information bias, interviews with all of the participants were conducted without disclosing their disease status after endoscopic examination. In addition, to avoid misclassification bias, controls were recruited from the same hospitals within 1 year and were confirmed to have no gastric problems on gastroscopy. However, this study also has certain limitations. First, the cases were recruited from only two hospitals, while the controls were recruited from the same hospitals; thus, they may be less representative of the general population. However, despite this limitation, there are the following advantages of recruitment control in hospitals [28, 29]; 1) it is easier to recruit and more likely to have similar quality of medical records will be comparable. 2) it is important to have high comparability in case-control studies. It has the advantage of increasing comparability with the case group in various characteristics. 3) recruitment of cases and controls under similar conditions can reduce information bias. Second, there may also be unintentional non-differential misclassifications because of a slight difference in the content of the FFQ survey depending on the recruitment period. Third, we adjusted for various confounding factors in the statistical model; however, there may have been residual confounders. Forth, in our study, the *H. pylori* infection rate was found to be lower in the cases than in the controls. This might be since *H. pylori* detection rates can decrease with the progression of gastric atrophy and intestinal metaplasia [30, 31], which is known as the main precursor lesion of GC [32]. In addition, it is possible that the use of antibiotics in the cases caused a decrease in *H. pylori* detection. Fifth, we found that there was a significant lower the GC risk in men with overweight and obese. Weight loss is known as one of the cancer cachexia commonly observed in cancer patients [33]. Indeed, in a recent case-control study, the cases had significantly lower BMI than the controls [34]. Sixth, given the change in eating habits over time, the backwardness of the recruited sample may be a limitation of our study However, according to the Korea National Health and Nutrition Examination Survey, the intake of soy products (36.65g in 2008 to 33.62g in 2019) hardly changed [35, 36].

In summary, we found that men with the A allele of *MAP3K1* rs252902 had a higher the GC risk. In particular, this association was more pronounced in men with a low intake of soy products. Therefore, nutrition education is needed to practice healthy dietary habits that increase the intake of soy products and reduce the intake of salty foods, especially in men who have the risk allele A of *MAP3K1* rs252902, because of the increased risk of GC.

## Supplementary Information

**Additional file 1: Supplementary Table 1.** Associations of MAP3K1 rs252902 polymorphism with GC incidence by intakes of soy products in women aged 50 year and above.

## Data Availability

Information related to the MAP3K1 SNP analyzed in this study is provided by dbSNP (https://www.ncbi.nlm.nih.gov/snp/rs252902, Gene ID: 4214). The data presented in this study are available upon request from the corresponding authors. The data are not publicly available because of the privacy concerns of the subjects.

## Abbreviations

GC: gastric cancer
MAP3K1: mitogen-activated protein kinase kinase 1
NF-κB: nuclear factor kappa B
FFQ: food frequency questionnaire
MAPK: mitogen-activated protein kinase
MAF: minor allele frequency
HWE: Hardy–Weinberg equilibrium
