# Supplementary material for: Sex-dependent associations between MAP3K1 gene polymorphisms and soy products with the gastric cancer risk in Korea: a case-control study
Source: BMC Gastroenterol. 2022 Dec 12;22:513. doi: 10.1186/s12876-022-02569-3 (PMC9743679; doi:10.1186/s12876-022-02569-3)
Supplement: Supplementary file 1 — Additional file 1: Supplementary Table 1. Associations of MAP3K1 rs252902 polymorphism with GC incidence by intakes of soy products in women aged 50 year and above. [file 12876_2022_2569_MOESM1_ESM.docx]

Supplementary table 1. Associations of MAP3K1 rs252902 polymorphism with GC incidence by intakes of soy products in women aged 50 year and above

|  | **Women aged 50 year and above** | | |
| --- | --- | --- | --- |
| **Variables** | **No. of cases/controls** | **OR** | **(95 % CI)**^1)^ |
| Dominant model |  |  |  |
| GG | 22/19 | 1.00 | Ref. |
| GA+AA | 29/32 | 0.66 | (0.24-1.76) |
| Total intakes of Soy products (g/day) | |  |  |
| Low | 36/25 | 1.00 | Ref. |
| High | 15/26 | 0.37 | (0.13-1.04) |
| Low intakes of soy products |  |  |  |
| GG | 18/13 | 1.00 | Ref. |
| GA+AA | 18/12 | 1.61 | (0.38-6.83) |
| High intakes of soy products |  |  |  |
| GG | 4/6 | 1.00 | Ref. |
| GA+AA | 11/20 | 0.37 | (0.05-2.60) |

OR, odds ratios; CI, confidence interval.

^1)^Adjusted for age, sex, body mass index (≤ 22.99, or ≥ 23.0), education level (≤ middle school, ≥ high school, or missing), family history of gastric cancer (no or yes), smoking status (never, past and current smokers), alcohol consumption (never, past and current), hospital (Chungnam university hospital or Hanyang university Guri hospital), H. pylori infection (negative, positive, or undetermined), daily intakes of noodles and dumpling, and total energy intake (continuous).
